# Serological Response Predicts Normalization of Cerebrospinal Fluid Abnormalities at Six Months after Treatment in HIV-Negative Neurosyphilis Patients

**DOI:** 10.1038/s41598-017-10387-x

**Published:** 2017-08-30

**Authors:** Yao Xiao, Man-Li Tong, Li-Rong Lin, Li-Li Liu, Kun Gao, Mei-Jun Chen, Hui-Lin Zhang, Wei-Hong Zheng, Shu-Lian Li, Hui-Ling Lin, Zhi-Feng Lin, Tian-Ci Yang, Jian-Jun Niu

**Affiliations:** 1 Zhongshan Hospital, Medical College of Xiamen University, Xiamen, Fujian Province China; 2 Xiamen Hospital of Traditional Chinese Medicine, Xiamen, Fujian Province China; 3 Institute of Infectious Disease, Medical College of Xiamen University, Xiamen, Fujian Province China; 4 Xiamen Zhongshan Hospital, Fujian Medical University, Xiamen, Fujian Province China; 5 Xiamen Huli District Maternity and Child Care Hospital, Xiamen, Fujian Province China

## Abstract

This study aimed to determine whether a serological response could predict the normalization of cerebrospinal fluid (CSF) abnormalities at 6 months after treatment in human immunodeficiency virus (HIV)-negative neurosyphilis patients. A total of 123 neurosyphilis patients were recruited at baseline, 58 of these patients undergoing treatment, repeated CSF examinations and serological tests for syphilis at 6 months after treatment were included in the follow-up study. Before treatment, the CSF rapid plasma reagin (RPR) titer, CSF *Treponema pallidum* particle agglutination (TPPA) titer, CSF leukocyte count, and CSF protein concentration were correlated with both serum RPR and TPPA titers. At 6 months after treatment, 28 and nine patients achieved serological responses of RPR and TPPA tests, respectively. The sensitivities of the serological response of RPR and TPPA tests for identifying the normalization of CSF abnormalities were 60.0∼83.3% and 17.1~22.2%, respectively; and 75.0∼91.3% of patients showing serological response of RPR test also achieved CSF normalization, suggesting that the serological response could predict CSF normalization to some degree. Particularly, in patients with ≥8-fold decreases in the serum RPR titer, the CSF RPR, CSF leukocyte count, and CSF protein concentration had normalized, and follow-up lumbar puncture could be reduced considering the resolution of neurological symptoms.

## Introduction

Neurosyphilis refers to infection of the central nervous system by *Treponema pallidum*, which may occur at any stage of infection. Conventionally, the diagnosis of neurosyphilis is based on a reactive non-treponemal test (e.g., venereal disease research laboratory [VDRL] test or rapid plasma reagin [RPR] test), pleocytosis and/or elevated protein in cerebrospinal fluid (CSF) collected by lumbar puncture. In recent years, the serological activity of the RPR test was found to be associated with the development of neurosyphilis. In both human immunodeficiency virus (HIV)-infected and HIV-uninfected syphilis patients, those with higher serum RPR titer were reported to have a higher likelihood of neurosyphilis^1^. Therefore, the results of serum RPR tests have been recommended as predictors of neurosyphilis to avoid unnecessary lumbar puncture^1, 2^.

In addition to the diagnosis of neurosyphilis, the efficacy of neurosyphilis treatment is mainly assessed by CSF examination. A CSF examination is recommended to be repeated every six months to evaluate changes in CSF parameters and determine the need for retreatment^3^. However, performing a lumbar puncture in a clinical setting is logistically difficult, and patients often refuse the procedure. Thus, there is an urgent need to find a surrogate for the normalization of CSF abnormalities after neurosyphilis treatment. Recently, researchers found that a 4-fold decrease in the serum RPR titer or reversion of the test to a nonreactive status could be a strong indicator of a successful neurosyphilis treatment^4^. In that study, >90% of patients demonstrating normalization of the serum RPR titer also showed normalization of clinical and CSF abnormalities, with the exception of the CSF protein concentration, at 13 months after treatment. However, 78% of the study participants had HIV infection and therefore presented characteristics different from HIV-negative syphilis patients^5^. Whether the HIV status influences the relationship between the serum RPR titer and treatment success remains unclear. Additionally, although treponemal tests should not be used to assess disease activity and treatment outcomes^6^, our preliminary study found that the likelihood of neurosyphilis was positively associated with the reactivity of serum *Treponema pallidum* particle agglutination (TPPA) tests^7^. In the present study, we assessed whether the serological response of the RPR and TPPA tests could help predict the normalization of CSF abnormalities after treatment in HIV-negative neurosyphilis patients.

## Results

### Clinical characteristics of study participants

The baseline characteristics of 123 neurosyphilis patients are shown in Table 1. The median age of the participants was 52 years (interquartile range [IQR], 46–58 years), and the patients included 97 males (78.9%) and 26 females (21.1%). Before treatment, all participants had reactive baseline serum RPR (median titer, 1:16; IQR, 1:4–1:64) and serum TPPA tests (median titer, 1:10240; IQR, 1:2560–1:20480). Eighty-six (69.9%) and 114 (92.7%) patients had reactive baseline CSF RPR and CSF TPPA results, respectively. The median titers of the CSF RPR and CSF TPPA were 1:2 and 1:1280, respectively. Eighty-five (69.7%) and 67 (55.4%) patients had abnormal baseline CSF leukocyte count and CSF protein concentration, respectively. In this study, the most common stage of neurosyphilis was general paresis (49.6%), followed by meningovascular neurosyphilis (22.8%), syphilitic meningitis (16.3%), tabes dorsalis (7.3%) and asymptomatic neurosyphilis (4.1%).

**Table 1:** Clinical characteristics of the study participants.

Characteristics	Neurosyphilis at baseline (n = 123)	Neurosyphilis enrolled in follow-up (n = 58)
Sex ratio (male: female)	97:26	47:11
Age, median years (IQR)	52 (46–58)	50 (44–57)
Baseline serum RPR titer,median (IQR)	1:16 (1:4–1:64)	1:32 (1:16–1:64)
≥1:1, n (%)	123 (100)	58 (100)
Baseline serum TPPA titer, median (IQR)	1:10240 (1:2560–1:20480)	1:10240 (1:5120–1:20480)
≥1:80, n (%)	123 (100)	58 (100)
Baseline CSF RPR titer, median (IQR)	1:2 (negative-1:4)	1:2 (1:1–1:4)
≥1:1, n (%)	86 (69.9)	45 (77.6)
Baseline CSF TPPA titer, median (IQR)	1:1280 (1:320–1:10240)	1:2560 (1:320–1:10240)
≥1:80, n (%)	114 (92.7)	54 (93.1)
Baseline CSF leukocyte count, median (IQR)	21 (6–54)	18 (10–56)
>10 cells/μL, n (%)	85 (69.7)	43 (74.1)
Baseline CSF protein concentration, median (IQR)	572.1 (392.3–882.9)	632.0 (458.0–867.0)
>500 mg/L, n (%)	67 (55.4)	34 (58.6)
Clinical stages, n (%)		
Asymptomatic neurosyphilis	5 (4.1)	4 (6.9)
Syphilitic meningitis	20 (16.3)	8 (13.8)
Meningovascular neurosyphilis	28 (22.8)	13 (22.4)
General paresis	61 (49.6)	29 (50.0)
Tabes dorsalis	9 (7.3)	4 (6.9)

Abbreviations: IQR, interquartile range; RPR, rapid plasma reagin; TPPA, *T. pallidum* particle agglutination; CSF, cerebrospinal fluid.

### Correlations between CSF parameters and reactivity of serum RPR or TPPA before neurosyphilis treatment

The correlations between each CSF parameter and the serum RPR or TPPA titer were evaluated before neurosyphilis treatment (Table 2). Serum RPR titer was correlated with the CSF RPR titer (*r*_*s*_ = 0.604, *P* < 0.001), CSF TPPA titer (*r*_*s*_ = 0.464, *P* < 0.001), CSF leukocyte count (*r*_*s*_ = 0.336, *P* < 0.001) and CSF protein concentration (*r*_*s*_ = 0.243, *P* = 0.007). Similarly, the serum TPPA titer was correlated with the CSF RPR titer (*r*_*s*_ = 0.634, *P* < 0.001), CSF TPPA titer (*r*_*s*_ = 0.756, *P* < 0.001), CSF leukocyte count (*r*_*s*_ = 0.401, *P* < 0.001) and CSF protein concentration (*r*_*s*_ = 0.341, *P* < 0.001). Neither the serum RPR titer nor the serum TPPA titer was correlated with CSF albumin, CSF glucose, CSF chloride, CSF lactate or CSF lactate dehydrogenase.

**Table 2:** Correlations between CSF parameters and reactivity of serum RPR or TPPA before neurosyphilis treatment (n = 123).

CSF parameters	Serum RPR titer		Serum TPPA titer	
	*r*_*s*_	*P*	*r*_*s*_	*P*
CSF RPR titer	0.604	**<0.001**	0.634	**<0.001**
CSF TPPA titer	0.464	**<0.001**	0.756	**<0.001**
CSF leukocyte count	0.336	**<0.001**	0.401	**<0.001**
CSF protein concentration	0.243	**0.007**	0.341	**<0.001**
CSF albumin	0.093	0.429	0.219	0.063
CSF glucose	−0.168	0.065	−0.038	0.678
CSF chloride	0.125	0.173	−0.126	0.172
CSF lactate	0.162	0.169	0.190	0.108
CSF lactate dehydrogenase	−0.124	0.294	−0.088	0.457

Abbreviations: RPR, rapid plasma reagin; TPPA, *T. pallidum* particle agglutination; CSF, cerebrospinal fluid.

### Associations between normalization of CSF abnormalities and serological response of RPR or TPPA test at 6 months after neurosyphilis treatment

Fifty-eight neurosyphilis patients undergoing treatment, repeated CSF examinations and serological tests for syphilis at 6 months after treatment were included in the follow-up study. Fifty-three of these patients received the recommended penicillin therapy; the other five were allergic to penicillin and were treated with ceftriaxone or doxycycline. These patients were divided into early syphilis and late syphilis subgroups. There were only three patients in the early syphilis subgroup; of these, none achieved a serological response of RPR test, and one achieved a serological response of TPPA test. No patients had reactive baseline CSF RPR. Two patients had an abnormal baseline CSF leukocyte count, and one of them exhibited normalization at 6 months after treatment. One patient had an abnormal baseline CSF protein concentration and exhibited normalization. The early syphilis subgroup was not included in further statistical analyses due to the small sample size.

In the late syphilis subgroup, which consisted of 18 late syphilis patients and 37 patients with syphilis of unknown duration, 28 (50.9%) and eight (14.5%) patients achieved serological responses of RPR and TPPA tests, respectively. A ≥4-fold increase in the serum RPR or TPPA titer was not observed during the study period. Of 45 patients with reactive baseline CSF RPR, 26 (57.8%) achieved a response of CSF RPR test. Of 41 patients with an abnormal baseline CSF leukocyte count, 35 (85.4%) exhibited a normalized CSF leukocyte count. Of 33 patients with an abnormal baseline CSF protein concentration, 18 (54.5%) exhibited a normalized CSF protein concentration.

The associations between the normalization of CSF abnormalities and serological response of RPR or TPPA test were analyzed for the late syphilis subgroup. Among 26 patients with CSF RPR responses, 18 achieved a serological response of RPR test, and five achieved a serological response of TPPA test. The sensitivities of the serological responses of RPR and TPPA tests for identifying a response of CSF RPR were 69.2% and 19.2%, respectively (*P* = 0.001). Similarly, the sensitivities of the serological response of the RPR test for identifying normalization of the CSF leukocyte count and CSF protein concentration were 60.0% and 83.3%, respectively. These values were higher than the respective sensitivities of the serological response of TPPA test (60.0% vs. 17.1%, *P* < 0.001; 83.3% vs. 22.2%, *P* = 0.001; Table 3). Among 28 patients who achieved a serological response of RPR test, 22 patients had reactive baseline CSF RPR, and 18 (positive predictive value [PPV], 81.8%) achieved CSF RPR responses. The median decrease in the CSF RPR titer was 4-fold (IQR, 2-fold to 4-fold). Twenty-three patients had an abnormal baseline CSF leukocyte count, and 21 (PPV, 91.3%) exhibited a normalized CSF leukocyte count. The median decrease in the CSF leukocyte count was 28 cells/μL (IQR, 8–78 cells/μL). Twenty patients had an abnormal baseline CSF protein concentration, and 15 (PPV, 75.0%) exhibited a normalized CSF protein concentration. The median decrease in the CSF protein concentration was 353 mg/L (IQR, 299–463 mg/L). Among the eight patients who achieved a serological response of TPPA test, five of six (PPV, 83.3%) had CSF RPR responses, six of seven (PPV, 85.7%) had a normalized CSF leukocyte count, and four of five (PPV, 80.0%) had a normalized CSF protein concentration (Table 3).

**Table 3:** Sensitivities and positive predictive values of serological responses of RPR and TPPA tests for predicting normalization of CSF abnormalities at 6 months after neurosyphilis treatment.

	Response of CSF RPR test		Normalization of CSF leukocyte count		Normalization of CSF protein concentration	
	Sensitivity^†^ n/N (%)	PPV^‡^ n/N (%)	Sensitivity n/N (%)	PPV n/N (%)	Sensitivity n/N (%)	PPV n/N (%)
Serological response of RPR test	18/26 (69.2)	18/22 (81.8)	21/35 (60.0)	21/23 (91.3)	15/18 (83.3)	15/20 (75.0)
Serological response of TPPA test	5/26 (19.2)	5/6 (83.3)	6/35 (17.1)	6/7 (85.7)	4/18 (22.2)	4/5 (80.0)

^†^Sensitivity was calculated as the number of patients in whom both CSF abnormalities normalized and serum RPR titer responded divided by the sum of these patients and the number of patients in whom CSF abnormalities did normalize but serum RPR titer did not respond.

^‡^Positive predictive value (PPV) was calculated as the number of patients in whom both CSF abnormalities normalized and serum RPR titer responded divided by the sum of these patients and the number of patients in whom CSF abnormalities did not normalize but serum RPR titer did respond. PPV is the percentage of patients in whom response of serum RPR titer predicts normalization of CSF abnormalities.

### Varying decreases in serum RPR and TPPA titers in predicting normalization of CSF abnormalities at 6 months after neurosyphilis treatment

We further analyzed the CSF profiles of patients with varying decreases in serum RPR and TPPA titers for the late syphilis subgroup. Fourteen patients exhibited no decrease in the serum RPR titer. Among these patients, 12 had reactive baseline CSF RPR, and 25.0% (3/12) achieved CSF RPR responses. Furthermore, 77.8% had a normalized CSF leukocyte count, and 25.0% had a normalized CSF protein concentration. Fourteen patients had a 2-fold decrease, 19 patients had a 4-fold decrease, four patients had an 8-fold decrease, and four patients had a 16-fold decrease in the serum RPR titer. As the serum RPR titer decreased, the normalization of CSF abnormalities increased. In patients whose serum RPR titer decreased ≥8-fold, the CSF RPR, CSF leukocyte count, and CSF protein concentration had normalized (Table 4). Unlike the serum RPR titer, the serum TPPA titer did not decrease in 60.0% (33/55) of participants. Nonetheless, among patients who presented a 4-fold decrease in serum TPPA titer, 83.3%, 85.7% and 80.0% exhibited a CSF RPR response, normalization of the CSF leukocyte count and normalization of the CSF protein concentration, respectively.

**Table 4:** Varying decreases in serum RPR and TPPA titers in predicting normalization of CSF abnormalities at 6 months after neurosyphilis treatment.

	Response of CSF RPR test n/N^a^ (%)	Normalization of CSF leukocyte count n/N^b^ (%)	Normalization of CSF protein concentration n/N^c^ (%)
**Decrease in serum RPR titer**			
Null (14 patients)	3/12 (25.0)	7/9 (77.8)	2/8 (25.0)
2-fold (14 patients)	5/11 (45.5)	7/9 (77.8)	1/5 (20.0)
4-fold (19 patients)	11/15 (73.3)	15/17 (88.2)	10/15 (66.7)
8-fold (4 patients)	4/4 (100.0)	4/4 (100.0)	2/2 (100.0)
16-fold (4 patients)	3/3 (100.0)	2/2 (100.0)	3/3 (100.0)
**Decrease in serum TPPA titer**			
Null (33 patients)	12/28 (42.9)	20/24 (83.3)	10/19 (52.6)
2-fold (14 patients)	9/11 (81.8)	9/10 (90.0)	4/9 (44.4)
4-fold (8 patients)	5/6 (83.3)	6/7 (85.7)	4/5 (80.0)

Abbreviations: RPR, rapid plasma reagin; CSF, cerebrospinal fluid.

^a^45 patients with reactive baseline CSF RPR were included.

^b^41 patients with an abnormal baseline CSF leukocyte count (>10 cells/μL) were included.

^c^33 patients with an abnormal baseline CSF protein concentration (>500 mg/L) were included.

## Discussion

The success of neurosyphilis treatment is commonly determined by the resolution or stabilization of clinical abnormalities and the normalization of CSF abnormalities. However, clinical abnormalities are more likely to resolve in early neurosyphilis. In contrast, patients with dementia or tabes are unlikely to show reversal^8^. Therefore, CSF assessment is an objective method for monitoring the effectiveness of therapy. Typically, neurosyphilis patients are recommended to undergo repeated lumbar punctures every 6 months after treatment until the CSF profiles become normal^3^. In HIV-infected patients, Marra *et al*.^4^ observed that the odds of normalization of the CSF VDRL titer, CSF leukocyte count and CSF protein concentration after treatment were 57-, 41- and 28-fold higher, respectively, when the serum RPR titer had normalized than when it had not. These researchers noted that a reduction in the serum RPR titer could serve as a surrogate for the normalization of CSF and clinical abnormalities after neurosyphilis treatment. In the present study, we examined this possibility in an HIV-negative population and analyzed whether variations in serum TPPA can predict the normalization of CSF.

A high serum RPR titer has been reported to be associated with an increased likelihood of neurosyphilis^1^. An increase in the RPR titer can reflect recent infection, reinfection or relapse. Therefore, the titer level is considered to be generally correlated with disease activity^9^. In the present study, we observed correlations between the serum RPR titer and several CSF parameters (including CSF RPR titer, CSF TPPA titer, and CSF leukocyte count) in neurosyphilis patients before treatment. In contrast, treponemal tests are considered irrelevant to disease activity due to persistent positive results for life. As a quantitative treponemal test, the TPPA test is based on agglutination. The quantitation of treponemal antibody is possible but has not been demonstrated to be worthwhile^10^. However, we found that the serum TPPA titer was also correlated with those CSF profiles before treatment. Our results suggest that serological activity of both the RPR and TPPA tests could reflect CSF abnormalities before neurosyphilis treatment.

Furthermore, we determined whether seroreactivity continues to reflect the CSF profiles after neurosyphilis treatment. Among the 55 patients involved in this analysis, 28 achieved a serological response of RPR test, whereas only eight achieved a serological response of TPPA test at 6 months after treatment. A serological response of RPR test was more sensitive than a serological response of TPPA test for identifying CSF normalization, with sensitivities of 69.2%, 60%, and 83.3% for identifying normalization of the CSF RPR, CSF leukocyte count, and CSF protein concentration, respectively. These findings differ from those of a previous study^4^ with a cohort that included 78% HIV-infected individuals and showed sensitivities of >90% (calculated from the reported data) for identifying CSF normalization. This inter-study discrepancy might due to the impact of HIV infection. Before neurosyphilis treatment, HIV-infected patients had a higher baseline serum RPR titer and were 2.5-fold less likely to show normalization of CSF-VDRL reactivity than HIV-uninfected subjects after treatment^11^.

The leukocyte count is a sensitive measure of the effectiveness of therapy. The leukocyte count declines earlier than the CSF-VDRL or CSF protein concentration after therapy and determines the need for retreatment^3^. In our study, despite moderate sensitivity, 91.3% (21/23) of patients with a serological response of RPR test also achieved normalization of the CSF leukocyte count. Further analysis showed that as the serum RPR titer decreased from null to 16-fold, a higher proportion of patients achieved normalization of CSF abnormalities. Notably, among patients whose serum RPR titer had decreased ≥8-fold, the CSF RPR, CSF leukocyte count, and CSF protein concentration all normalized. Our results suggest that a serological response of RPR test could serve as a surrogate for the normalization of CSF abnormalities after neurosyphilis treatment and that repeated lumbar puncture could be reduced in patients with ≥8-fold reductions in the serum RPR titer considering the resolution of neurological symptoms. Conventionally, treponemal tests are not used for assessing treatment outcome^6^. In the present study, 60.3% (35/58) of neurosyphilis patients did not exhibit any change in the serum TPPA titer at 6 months after treatment, suggesting that the development of a serum TPPA response was a long process. Of interest, among those who exhibited a serological response of TPPA test, more than 80.0% also experienced normalization of CSF abnormalities. This finding warrants further consideration and research.

Our study is the first to examine the utility of the serological response in predicting the normalization of CSF abnormalities after neurosyphilis treatment in HIV-negative individuals. However, our findings might be limited due to the inadequate sample size. Only 47.15% (58/123) of neurosyphilis patients underwent treatment, repeated CSF examinations and serological tests for syphilis at 6 months after treatment, and only eight patients with ≥8-fold decreases in the serum RPR titer were analyzed. Fewer patients made return visits for follow-up at 12 months; accordingly, we failed to conduct a longer follow-up investigation. The baseline serum RPR, serum TPPA, and CSF RPR titer, CSF leukocyte count, and CSF protein concentration of the patients lost to follow-up were compared with those of the 58 study participants, and no significant differences were found. Although the loss to follow-up may have had little impact on the results, analysis of an expanded sample is needed.

Our study has practical implications for clinicians caring for HIV-negative neurosyphilis patients. Before neurosyphilis treatment, the serological activity of RPR and TPPA might reflect CSF abnormalities. After neurosyphilis treatment, a serological response of RPR test was more sensitive for predicting normalization of CSF abnormalities than a serological response of TPPA test. In HIV-negative neurosyphilis patients with ≥8-fold decreases in the serum RPR titer, the CSF RPR, CSF leukocyte count, and CSF protein concentration had all normalized, and follow-up lumbar puncture could be reduced considering the resolution of neurological symptoms.

## Methods

### Study population and ethics statement

**Figure 1:**
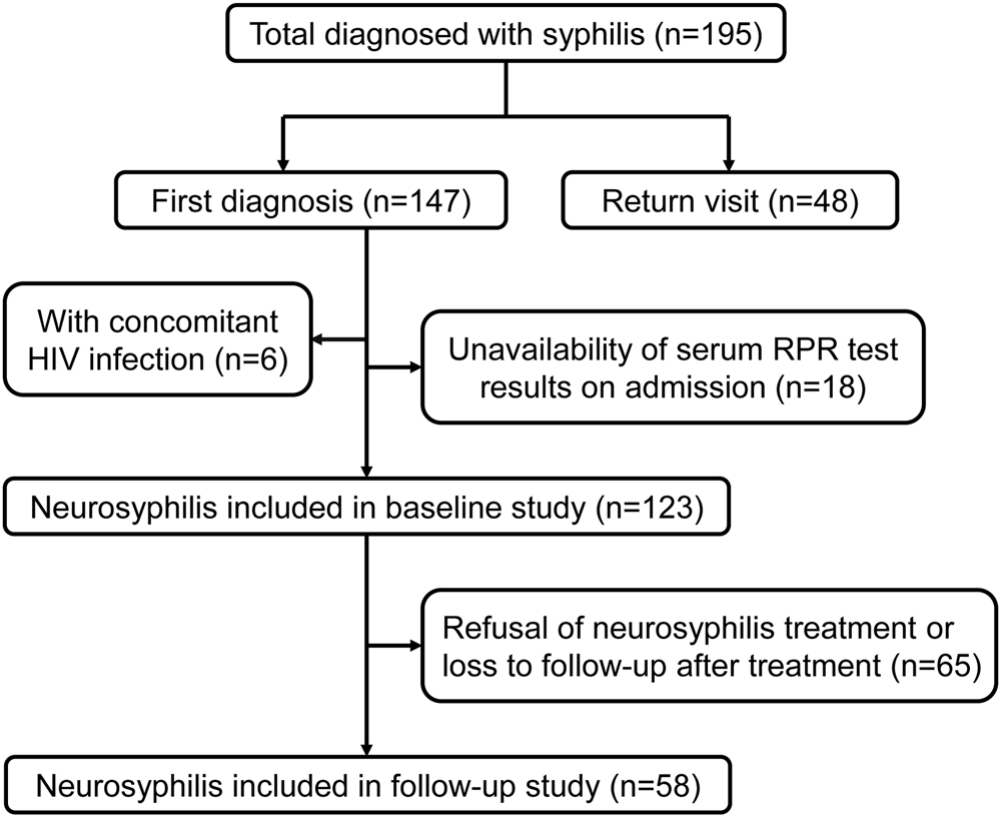
Flow chart of the participants from enrollment.

Between June 2008 and December 2014, 195 hospitalized patients were diagnosed with neurosyphilis based on clinical and laboratory findings at Zhongshan Hospital, Medical College of Xiamen University. After excluding returning patients, HIV-infected individuals, and patients with unavailable serum RPR test results on admission, 123 neurosyphilis patients were included in the baseline study. Eventually, 58 neurosyphilis patients undergoing treatment, repeated CSF examinations and serological tests for syphilis at 6 months after treatment were included in the follow-up study (Fig. 1). This study was approved by the Institutional Ethics Committee of Zhongshan Hospital, Medical College of Xiamen University, and was in compliance with national legislation and the Declaration of Helsinki guidelines. Written informed consent was obtained from all study subjects.

### Diagnostic criteria

The diagnostic criteria for neurosyphilis complied with the guidelines of the Centers for Disease Control in the USA and Europe^12, 13^ as used in our previous study^7, 14^. Neurosyphilis was defined as syphilis at any stage with one or more of the following findings: (1) a reactive RPR in CSF, (2) positive TPPA assay and elevated leukocyte count (>10 cells/μL) in CSF, and (3) elevated CSF protein concentration (>500 mg/L) and/or leukocyte count (>10 cells/μL) in the absence of other known causes of these abnormalities, and clinical symptoms or signs consistent with neurosyphilis without other known causes for these clinical abnormalities. Asymptomatic neurosyphilis was defined as syphilis at any stage that met the laboratory criteria for neurosyphilis but without clinical symptoms or signs. Neurosyphilis patients who acquired syphilis infection during the preceding year were staged as early syphilis; patients who had positive serological tests for syphilis for >1 year, or whose duration of syphilis infection was unknown, were staged as late syphilis^12^.

In this study, at 6 months after neurosyphilis treatment, a serological response of RPR or TPPA test was defined as a ≥4-fold (2 dilutions) decrease in titer (e.g., from 1:16 to 1:4 or from 1:5120 to 1:1280) or reversion of the test to nonreactive. A serological nonresponse was defined as a <4-fold decrease or any increase in titer. A response of CSF RPR test was defined as a ≥4-fold decrease in titer or reversion of the test to nonreactive. Normalization of the CSF leukocyte count was defined as a decrease from >10 cells/μL to ≤10 cells/μL. Normalization of the CSF protein concentration was defined as a decrease from >500 mg/L to ≤500 mg/L.

### Laboratory tests

The CSF examination and serological tests for syphilis were performed using RPR (InTec, Xiamen, China) and TPPA (Fujirebio, Tokyo, Japan) tests according to the manufacturers’ instructions and as previously reported^14–16^, as follows. Briefly, the samples were subjected to the RPR test with an 18-mm circular test card following a standardized method. The antigen suspension contained carbon particles coated with cardiolipin antigens. Approximately 50 μL of serum was dispensed over the card, followed by a drop of the antigen suspension. The card was placed on a rotating plate at 100 rpm for 8 min to mix the antigen suspension and serum. Readings were obtained immediately with the naked eye through comparisons with negative and positive controls. The serum samples that reacted to RPR were quantified using 2-fold serial dilutions. The TPPA assay is based on the agglutination of colored gelatin particles that have been sensitized (coated) with *T. pallidum* (Nichols strain) antigen. The initial dilution of the serum samples used in the TPPA reactions was 1:80. Two standard serum samples (including 400 mIU/mL and 80 mIU/mL) (Beijing Controls & Standards Biotechnology Co., Ltd., China) were used as controls for the RPR and TPPA reactions, respectively. Serum samples that produced conflicting or inconclusive results for a particular technique were tested again; the new result was considered the true result.

CSF samples (approximately 2 mL) were collected within a time frame of 2 h. Blood (5 mL) was collected in plain sterile tubes from patients who had fasted for at least 8 h. The blood was then centrifuged within 10 min and analyzed within 4 h. The protein, albumin, lactate, lactate dehydrogenase, chloride, and glucose levels in the CSF samples were measured using a Roche-Hitachi Modular P800 Analyzer (Roche Diagnostics, F. Hoffmann-La Roche, Ltd., Basel, Switzerland). The CSF leukocyte count was measured using an Automatic Blood Cell XE5000 Analyzer (Sysmex International Reagents, Co., Ltd, Japan).

### Statistical analysis

Spearman’s rank correlation was used to analyze the correlations between each of the CSF parameters and the reactivity of both serum RPR and serum TPPA. Serum RPR and serum TPPA, which are quantitative measures with serial 2-fold dilutions, were log_2_ transformed for the correlation analysis. McNemar’s test was used to compare the sensitivities of the serological responses of RPR and TPPA tests for identifying the normalization of CSF abnormalities. The statistical analyses were performed using SPSS 19.0 for Windows (SPSS Inc., Chicago, IL, USA). A two-sided *P*-value of <0.05 was considered statistically significant.

## References

[1] Marra CM (2004). Cerebrospinal fluid abnormalities in patients with syphilis: Association with clinical and laboratory features. J Infect Dis.

[2] Shi M (2015). Risk profiles of neurosyphilis in HIV-negative patients with primary, secondary and latent syphilis: Implications for clinical intervention. J Eur Acad Dermatol Venereol.

[3] Workowski KA, Bolan GA (2015). Sexually transmitted diseases treatment guidelines, 2015. MMWR Recomm Rep.

[4] Marra CM, Maxwell CL, Tantalo LC, Sahi SK, Lukehart SA (2008). Normalization of serum rapid plasma reagin titer predicts normalization of cerebrospinal fluid and clinical abnormalities after treatment of neurosyphilis. Clin Infect Dis.

[5] Knaute DF, Graf N, Lautenschlager S, Weber R, Bosshard PP (2012). Serological response to treatment of syphilis according to disease stage and HIV status. Clin Infect Dis.

[6] Janier M (2014). European guideline on the management of syphilis. J Eur Acad Dermatol Venereol.

[7] Xiao Y (2017). Novel predictors of neurosyphilis among HIV-negative syphilis patients with neurological symptoms: An observational study. BMC Infect Dis.

[8] Marra CM (2015). Neurosyphilis. Continuum (Minneap Minn).

[9] Lin LR (2011). Further evaluation of the characteristics of Treponema pallidum-specific IgM antibody in syphilis serofast reaction patients. Diagn Microbiol Infect Dis.

[10] Larsen SA, Steiner BM, Rudolph AH (1995). Laboratory diagnosis and interpretation of tests for syphilis. Clin Microbiol Rev.

[11] Marra CM (2004). Normalization of cerebrospinal fluid abnormalities after neurosyphilis therapy: Does HIV status matter?. Clin Infect Dis.

[12] Centers for Disease Control and Prevention. STD surveillance case definitions. *Centers for Disease Control and Prevention*. http://www.cdc.gov/std/stats/CaseDefinitions-2014.pdf (2014).

[13] French P (2009). IUSTI: 2008 European Guidelines on the Management of Syphilis. Int J STD AIDS.

[14] Liu LL (2013). Assessing cerebrospinal fluid abnormalities in neurosyphilis patients without human immunodeficiency virus infection. Int Immunopharmacol.

[15] Xiao Y (2016). Factors associated with syphilis infection: A comprehensive analysis based on a case-control study. Epidemiol Infect.

[16] Liu F (2014). Characterization of the classical biological false-positive reaction in the serological test for syphilis in the modern era. Int Immunopharmacol.

